# Land cover classification of high-resolution remote sensing images based on improved spectral clustering

**DOI:** 10.1371/journal.pone.0316830

**Published:** 2025-02-06

**Authors:** Song Wu, Jian-Min Cao, Xin-Yu Zhao

**Affiliations:** Jilin Agricultural University, Changchun, China; COMSATS University Islamabad, PAKISTAN

## Abstract

Applying unsupervised classification techniques on remote sensing images enables rapid land cover classification. Using remote sensing imagery from the ZY1-02D satellite’s VNIC and AHSI cameras as the basis, multi-source feature information encompassing spectral, edge shape, and texture features was extracted as the data source. The Lanczos algorithm, which determines the largest eigenpairs of a high-order matrix, was integrated with the spectral clustering algorithm to solve for eigenvalues and eigenvectors. The results indicate that this method can quickly and effectively classify land cover. The classification accuracy was significantly improved by incorporating multi-source feature information, with a kappa coefficient reaching 0.846. Compared to traditional classification methods, the improved spectral clustering algorithm demonstrated better adaptability to data distribution and superior clustering performance. This suggests that the method has strong recognition capabilities for pixels with complex spatial shapes, making it a high-performance, unsupervised classification approach.

## 1. Introduction

Land cover classification provides technical support for land planning and management, land change mechanism analysis, and environmental protection. With its macroscopic, dynamic, and rapid characteristics, remote sensing technology has become the most effective means of obtaining land use information [1, 2]. The automatic classification of land cover and thematic information extraction using satellite remote sensing data has long been at the forefront of remote sensing technology applications [3]. In recent years, more studies have utilized high-resolution remote sensing data to achieve automatic land cover classification, yielding significant results [4–6]. Traditional unsupervised remote sensing image classification methods are relatively easy to implement and have lower computational complexity. These methods perform well when pixel clusters exhibit a simple probability distribution in spectral space and point clusters in remote sensing images have convex geometric shapes [7]. Consequently, traditional unsupervised methods often rely on high-resolution data for remote sensing image classification. However, these methods are primarily limited to extracting and analyzing shape and texture features, resulting in constrained classification accuracy. When there are significant differences in pixel counts between clusters or when the pixel sets comprising these clusters do not follow a Gaussian distribution, classification performance deteriorates noticeably [8–10]. In such cases, shape features perform poorly in complex terrains, and relying solely on texture features fails to capture surface objects’ spectral information adequately [11–13]. As a result, multi-feature fusion methods that integrate shape, texture, and spectral information have increasingly become a research focus [14]. These methods effectively utilize shape features to capture the geometric structure of land objects, texture features to represent surface roughness, and spectral features to provide information about physical and chemical composition [15–17]. By leveraging these combined features, classification accuracy and robustness can be significantly enhanced, addressing the limitations of single-feature classification methods [18].

In remote sensing image classification, unsupervised classification methods offer significant advantages over supervised methods. Unsupervised classification does not require pre-labeled training data, which is particularly important when labeled data is expensive or difficult to obtain [19]. Additionally, unsupervised methods can adaptively discover underlying structures and patterns in the data, making them suitable for complex and unknown data distributions. Among various unsupervised classification methods, spectral clustering models demonstrate unique advantages. Spectral clustering constructs a data similarity matrix and applies spectral graph theory to perform classification. It can identify non-convex clusters and does not require assumptions about the global structure of the data [20]. Compared to traditional clustering algorithms such as the Iterative Self-Organizing Data Analysis Technique (ISODATA) and k-means, spectral clustering exhibits greater adaptability to data distribution. It has proven effective in high-resolution remote sensing image processing. Traditional methods like k-means are generally more effective for spherical clusters, whereas spectral clustering excels at handling non-convex clusters. By relying on the global similarity structure rather than the distribution of locally adjacent points, spectral clustering demonstrates high robustness to noise and outliers. Consequently, spectral clustering has become a significant research topic in the field of machine learning [21].

In recent years, numerous scholars have conducted extensive research on the theory and application of spectral clustering, yielding a series of significant results. Theoretically, researchers have proposed several improved spectral clustering algorithms to address the limitations of traditional spectral clustering. Notable contributions include Recursive Spectral Clustering [22], Class-Specific Spectral Clustering [23], Fuzzy Spectral Clustering [24, 25], Mean Shift Spectral Clustering [26], Efficient Evolutionary Spectral Clustering [27], Sparse Kernel Spectral Clustering [28], Fast Kernel Spectral Clustering [29], Non-negative Sparse Spectral Clustering [30], Vector Quantization-based Approximate Spectral Clustering [31], and Compressed Constraint Spectral Clustering [32]. In terms of applications, spectral clustering has been successfully applied to various fields, including face recognition [33], image segmentation [34], big data analysis and processing [35], medical image analysis [36], information retrieval [37], power system modeling [38], protein data analysis [39], and disaster warning systems [40].

Although significant progress has been made in the theory and application of spectral clustering, there has been relatively little research on the unsupervised classification of remote sensing images using spectral clustering. Moreover, most improved Spectral Clustering (SC) algorithms rely on distance metrics such as Euclidean distance, cosine distance, or Gaussian kernel distance, which often fail to capture the topological structure of the data. This study introduces the adaptive spectral clustering algorithm combining the shared proximity and flow distance (SNN-MSC) approach to the field of remote sensing image processing, using high-resolution remote sensing images as the data source. By integrating spectral, edge shape, and texture information, and replacing traditional distance metrics used in spectral clustering with manifold distance, the density factor is incorporated to impose additional constraints on the similarity matrix. This optimization enhances the spectral clustering algorithm for unsupervised remote sensing image classification, enabling effective land cover classification of high-resolution remote sensing imagery. Additionally, through research on automatic land cover classification, this study tests the applicability of spectral clustering algorithms to large-scale data clustering problems, aiming to address the limitations of current algorithms and further develop the theoretical foundations of spectral clustering.

## 2. Materials and methods

### 2.1 Study area and data resource

**Fig 1:**
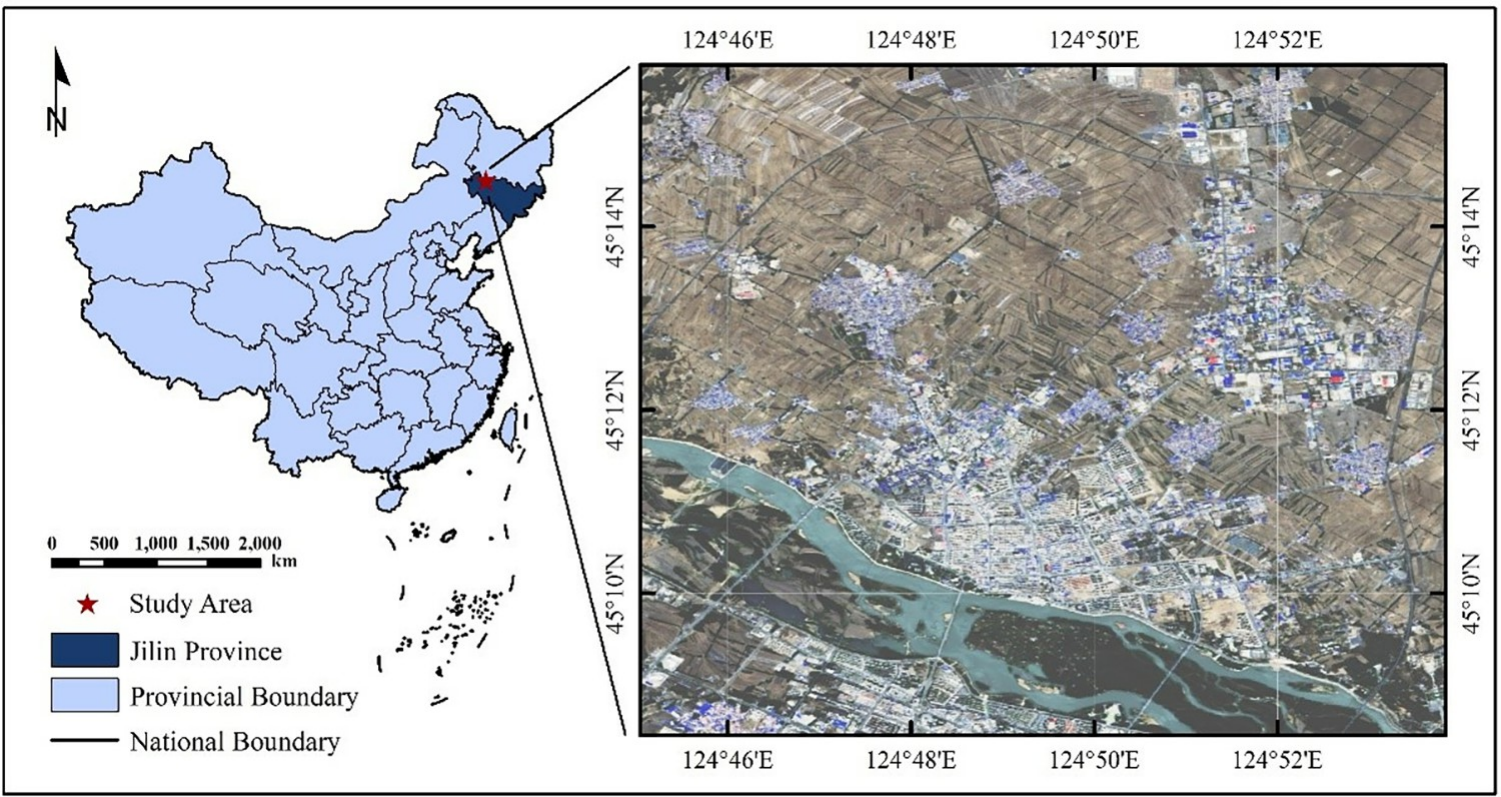
Study area for land cover classification in Songyuan City, Jilin Province, China.

The study area is located within Songyuan City, in the central-western region of Jilin Province, China. It lies within the Harbin-Changchun-Daqing triangle, at the core of the Ha-Chang urban agglomeration, and serves as a crucial transportation hub and logistics distribution center in Northeast China (Fig 1). Situated in the mid-latitude region of the Northern Hemisphere, the study area experiences a temperate continental monsoon climate, characterized primarily by plains. The average annual temperature is approximately 4.5°C, with total annual precipitation ranging from 400 mm to 500 mm. The study area in Antu County is located in southwest of Yanbian Prefecture, Jilin Province, China. It is at the northern foot of Changbai Mountain, where landform is mainly mountains, with warm climate, abundant rainfall and resources. It was a famous hometown of Chinese mineral water and a breeding base for Chinese herbal medicines. In this study, Gaofen-2 (GF-2) satellite images obtained on May 21, 2017 are used (https://www.cresda.com, Authorized). GF-2 is the first satellite launched by Chinese high resolution Earth Observation System. It is equipped with 1 m panchromatic and 4 m multispectral cameras to achieve imaging, and has the meter-level spatial resolution, high radiation and positioning accuracy, fast revisiting cycle. GF-2 images have been widely applied in land resource survey and monitoring. A typical area with rich land-cover types in the study area is selected, corresponding to the 400×400 pixel range of the image. The land-cover types in the area mainly include cropland, forest, grassland, building and water, which can effectively test the classification method. This study utilized ZY1-02D satellite images acquired on May 10, 2023. The ZY1-02D satellite, also known as Resource One 02D, is equipped with both visible-near infrared and hyperspectral cameras, enabling the simultaneous acquisition of detailed texture and rich spectral information of land features (https://www.sasclouds.com, Authorized). Compared to other satellites, the ZY1-02D offers a more comprehensive description of land characteristics and a broader single-pass coverage, making it particularly advantageous for urban and rural planning as well as resource monitoring. A typical area within the study region, characterized by diverse land cover types, was selected, corresponding to an image range of 400 × 400 pixels. The land cover types in this area include water bodies, croplands, forests, buildings, and transportation infrastructure, which are well-suited for evaluating the classification methods.

### 2.2 SNN-MSC algorithm

Spectral clustering is a prominent deep-learning algorithm based on graph theory, a branch of mathematics focused on the study of graphs. Graph partitioning theory treats different sample points as vertices of a graph, connects each pair of vertices with an edge, and constructs a spectral graph according to specific rules. Similarity between samples is used to assign weights to the edges, resulting in a weighted undirected graph based on sample similarity, thus transforming the clustering problem into a graph partitioning problem [41, 42]. The construction of the similarity matrix directly impacts the accuracy of spectral clustering (SC) algorithms [43]. Traditional SC algorithms often use distance metrics such as Euclidean distance, cosine distance, or Gaussian kernel distance. These metrics typically fail to capture the topological structure of the data, leading to neglect of global consistency and insufficient capture of the data’s intrinsic structure, which results in suboptimal clustering outcomes [44]. The SNN-MSC algorithm introduces a novel manifold distance with exponential terms and proportional factors. By adjusting these terms, the algorithm modifies the similarity ratio between data points within the same manifold and those across different manifolds, thus preserving global and local data distribution consistency. Additionally, the algorithm incorporates a density factor to mitigate noise effects and computes similarity based on the sparsity and density of data neighborhoods, enhancing the neighborhood information between points. Rank constraints are applied to the Laplacian to ensure that the number of connected components in the similarity matrix equals the number of clusters.

First, by integrating the k-nearest neighbors of the samples with the mean level of the dataset’s neighborhood information, the density factor is defined as follows:

$$\rho_{i}=e^{\left(\omega \times (\xi /d_{avg}(x_{i}))\right)},\forall i=1,2,\cdots ,n$$ (1)

Where *ρ*_*i*_ denotes the density factor of sample *x*_*i*_, with larger values indicating higher local density. *ω*​ represents the weight, which is set to 1 in this study. *k* signifies the number of nearest neighbors, and *d*_*avg*_(*x*_*i*_)​ is the average Euclidean distance between sample *x*_*i*_ and its *k* nearest neighbors. A smaller *d*_*avg*_(*x*_*i*_)​ value implies that sample *x*_*i*_ is closer to its *k* nearest neighbors, indicating a higher local density. *ξ* represents the mean level of overall neighborhood information within the dataset. *KNN*(*x*_*i*_)​ is the set of *k* nearest neighbors of sample *x*_*i*_, and *θ*​ denotes the distance to the *k-th* nearest neighbor from sample *x*_*i*_.

The distance between samples is calculated by incorporating the k-nearest neighbor information from the original space to compute the density factor, which is then integrated into the manifold distance metric. This redefines the distance between samples, as shown in Eq (2):

$$D_{ij}^{M}=\frac{1}{\theta }\ln(1+\underset{p\in P_{ij}}{\min})\overset{\left|p\right|}{\underset{a-1}{\sum }}(e^{\theta d(p_{a,}p_{a+1})/(\rho_{a}\rho_{a+1})}-1))$$ (2)

Where $D_{ij}^{M}$ denotes the distance between samples *x*_*i*_ and *x*_*j*_; *θ*>0 represents the scaling factor, where a smaller *θ* value makes the corrected distance metric more inclined towards local consistency, while a larger *θ* value favors global consistency. *p* denotes a path of length *l*=|*p*|−1, |*p*| represents the length of the path *p* connecting samples *x*_*i*_ and *x*_*j*_, and (*p*_*a*_,*p*_*a*+1_)∈*E* represents the short edge formed by two adjacent points *p*_*a*_ and *p*_*a*+1_ on the path *p*. *P*_*ij*_ indicates the set of all paths connecting samples *x*_*i*_ and *x*_*j*_, *d*(*p*_*a*_,*p*_*a*+1_) denotes the Euclidean distance between any two adjacent nodes on the path, and $\underset{p\in P_{ij}}{\min}\sum_{a-1}^{\left|p\right|}\left(e^{\theta d(p_{a},p_{a+1})/(\rho_{a}\rho_{a+})}-1\right)$ is the minimum path distance between samples *x*_*i*_ and *x*_*j*_ on graph *G*. Finally, *ρ*_**a**_>0​ and *p*_**a**+1_>0​ represent the density factors of two adjacent nodes *p*_*a*_ and *P*_*a*+1_, respectively.

In the same manifold, sample points are typically connected by multiple short edges, whereas sample points from different manifolds require longer edges for connection. In this study, the manifold distance $e^{\theta d(p_{a},p_{a+1})/(\rho_{a}\rho_{a+1})}$​ is used in place of the traditional Euclidean distance *d*(*p*_*a*_,*p*_*a*+1_)​ to measure the distance between adjacent points. Additionally, the density factor is calculated by incorporating the neighborhood information of the samples, which helps to mitigate the impact of noise.

The regularization parameter is calculated using the following equation:

$$r_{i}=\frac{k}{2}D_{i,k+1}^{M}-\frac{1}{2}\sum_{j-1}^{k}D_{ij}^{M}$$ (3)

The mean value of *r*_1_,*r*_2_,⋯*r*_*n*_ is selected:

$$r=\frac{1}{n}\sum_{i-1}^{n}\left(\frac{k}{2}D_{i,k+1}^{M}-\frac{1}{2}\sum_{j-1}^{k}D_{ij}^{M}\right)$$ (4)

Using the number of shared neighbors and distances, combined with local scale information, the similarity *sim*_*ij*_ between sample *x*_*i*_ and sample *x*_*j*_ is redefined in the form of an exponential kernel, as follows:

$$sim_{ij}=\left\{ \begin{array}{l} e^{-\frac{\sum_{b\in SNN(x_{i},x_{j})}{\left(D_{ib}^{M}+D_{jb}^{M}\right)}^{2}}{\sigma_{i}\sigma_{j}(|SNN(x_{i},x_{j})|+1)}}i=j\\ 0\;i\neq j \end{array}\right.$$ (5)

Where *SNN*(*x*_*i*_,*x*_*j*_)​ represents the intersection of the k-nearest neighbor sets of samples *x*_*i*_ and *x*_*j*_, i.e., *SNN*(*x*_*i*_,*x*_*j*_) = *KNN*′(*x*_*i*_)∩*KNN*′(*x*_*j*_)​. Additionally, $D_{ik}^{M}$​ denotes the distance from sample *x*_*i*_ to its k-th nearest neighbor. The quantity |*SNN*(*x*_*i*_,*x*_*j*_)| indicates the number of shared neighbors between samples *x*_*i*_ and *x*_*j*_; a higher value reflects a greater number of shared neighbors and thus a higher similarity. *σ*_*i*_​ and *σ*_*j*_​ represent the local scales of samples *x*_*i*_ and *x*_*j*_, respectively, with values corresponding to the distance to the k-th nearest neighbor. Smaller values indicate higher local density, and the local scale can be adaptively adjusted based on neighborhood information, increasing the similarity between samples in sparse clusters to facilitate their aggregation and mitigate the limitations of global scale. The term $\sum_{b\in SNN(x_{i},x_{j})}{\left(D_{ib}^{M}+D_{jb}^{M}\right)}^{2}$ represents the sum of the squared distances of samples *x*_*i*_ and *x*_*j*_ to the points in the shared neighbor set​. Smaller values suggest that the two samples are relatively close, indicating higher similarity. In summary, a higher value of *sim*_*ij*_​ signifies a higher similarity between samples *x*_*i*_ and *x*_*j*_.

The calculated similarity matrix is not a normalized matrix; it needs to be normalized to obtain *S*. The formula is as follows:

$$s_{ij}=\frac{sim_{ij}}{\sum_{j-1}^{n}sim_{ij}}$$ (6)

It is commonly assumed that samples that are closer to each other have greater similarity. At the same time, to avoid trivial solutions, where only the closest samples are considered as neighbor points, a regularization term is introduced. If the similarity matrix s*s* is non-negative, the number of eigenvalues equal to zero in the corresponding Laplacian matrix corresponds to the number of connected components in the undirected graph *G*. Therefore, this paper imposes a rank constraint on the Laplacian matrix, with the objective function presented in Eq (7):

$$\underset{s}{\min}\sum_{i,j=1}^{n}(D_{ij}^{M}s_{ij}+\gamma {s_{ij}}^{2})$$ (7)

### 2.3 Feature extraction

#### 2.2.1 Image spectral feature extraction

**Fig 2:**
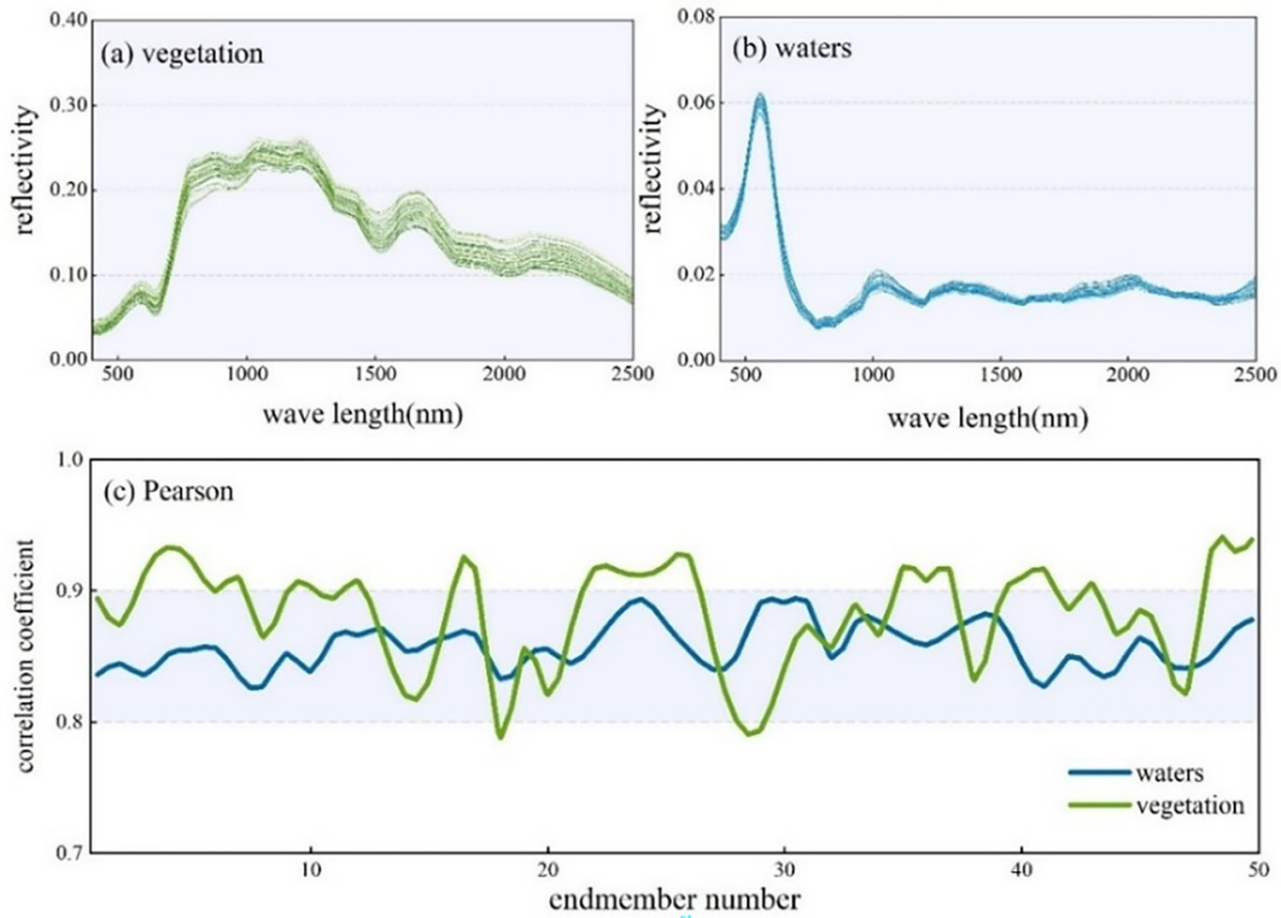
Endmember spectra and Pearson correlation coefficient curves.

Due to the presence of striping artifacts in the shortwave infrared (SWIR) bands of the ZY1-02D satellite hyperspectral data, a "global de-striping" method was employed to correct these artifacts. Additionally, atmospheric water vapor absorption affects wavelengths in the 1350–1420 nm and 1820–1920 nm ranges, leading to insufficient imaging quality of the spectral data. Therefore, spectral channels within these ranges were excluded, and the remaining 153 spectral bands were combined and stored. Radiometric calibration was performed on the remaining bands to correct for errors caused by sensor noise, solar position, and angle variations. The FLASH model was then used for atmospheric correction to obtain surface reflectance. Considering the issues of hyperspectral data mixing and the difference in spatial resolution compared to panchromatic data, the extracted feature data were resampled to a uniform resolution of 10 meters. Subsequently, geometric correction was applied to register the spatial positions of the data, ensuring classification accuracy (Fig 2).

During the electromagnetic radiation transmission and acquisition process, the total radiance measured by the sensor is affected by atmospheric molecules, aerosol scattering, and water vapor absorption, which impairs the ability of the sensor to reflect the true spectral characteristics of surface objects. This significantly impacts the clarity and contrast of the images, severely hindering the application of remote sensing data. Therefore, reducing or eliminating atmospheric noise and enhancing image features to recover and obtain accurate surface spectral reflectance data has been a key focus for researchers domestically and internationally. To assess the usability of ZY1-02D hyperspectral images, the corrected spectral image was overlaid with the current land use map. Endmember spectra for forested areas and water bodies, with 50 samples each, were extracted and compared with field-measured spectral data. The correlation was analyzed using Pearson correlation coefficients to evaluate the calibration effectiveness of the hyperspectral images. As illustrated, the spectral reflectance curves of the pixels and the field-measured spectra generally show similar spectral shapes and characteristic absorption features. Most samples had Pearson correlation coefficients between 0.8 and 0.9. Waterbody correlations were generally higher, while vegetation correlation curves showed more variability, though the average still exceeded 0.8. This indicates a high degree of match, suggesting that the image pixels retain most of the surface’s spectral features and are suitable for rapid urban land use classification. Urban construction land and water bodies exhibit significant differences in spectral reflectance, facilitating effective differentiation of land cover types through spectral information. The SNN-MSC algorithm improves distance metrics by considering both global and local consistency. It achieves this by adjusting the exponent and scaling factor to simultaneously satisfy global and local consistency, thereby better uncovering the intrinsic structure of the data and ensuring the reliability of the final clustering results.

#### 2.2.2 Texture and edge feature extraction

Texture is related to the spatial distribution of intensity values in an image, reflecting surface variation, structure, and organization properties. Texture features are categorized into four types: statistical, model-based, signal processing, and structural. Among these, the Gray-Level Co-occurrence Matrix (GLCM) is widely used in texture feature classification [45]. Haralick et al. proposed 14 texture features using GLCM, which primarily involve statistical analysis of the spatial correlation of image gray levels to compute texture. This study selects eight commonly used features as texture feature data sources: Mean, Variance, Homogeneity, Contrast, Dissimilarity, Entropy, Angular Second Moment, and Correlation.

#### 2.2.3 Spectral feature selection

The Successive Projections Algorithm (SPA), introduced by Bregman in 1965, is a pre-variable selection technique that utilizes vector projection analysis to select the most significant vectors and subsequently extract a few characteristic wavelengths through model calibration [46]. The advantages of SPA lie in its ability to select variable combinations with minimal collinearity from the spectral matrix, thereby reducing model redundancy and enhancing both the stability and accuracy of the model. The specific steps are as follows:

*x*_*k*(0)_ denotes the initial iteration vector; N denotes the number of variables to be extracted; J denotes the number of columns of the spectral matrix. Randomly pick the *j*-th column in the spectral matrix and assign it to *x*_*j*_ denoted as *x*_*k*(0)_; the set of the remaining column vectors is denoted as s. Calculate the projection *P*_*xj*_ of *x*_*j*_ on the remaining column vectors, respectively. Extract the spectral wavelength *k*_(*n*)_ of the largest projection vector, such that *x*_*j*_ = *p*_*x*_,*j*∈*s*, *n* = *n*+1, if n<N, is calculated cyclically according to the following equation.


s={j,1≤j≤J,j∉{k(0),⋯k(n−1)}} (8)

$$P_{xj}=x_{j}-(x_{j}^{T}x_{k(n-1)})x_{k(n-1)}{\left({x_{k(n-1)}}^{T}x_{k(n-1)}\right)}^{-1},j\in s$$ (9)

$$k_{\left(n\right)}=\arg(\max(\|P(x_{j})\|),j\in s)$$ (10)

Finally, the extracted variables are {*x*_*k*(*n*)_ = 0,⋯,*N*−1}, respectively, k(0) and N in each cycle of the multiple linear regression model, through the interactive validation of a root-mean-square error value, according to its corresponding candidate subset, select the smallest RMSE value corresponding to the k(0) and N determined as the final optimal value.

### 2.4 Algorithm modeling

After the initial modeling and evaluation, parameter optimization of the model is usually required to improve the classification accuracy and model stability. The bandwidth parameters of the Gaussian kernel function are first adjusted to optimize the similarity matrix construction. Moreover, feature selection is carried out to improve the classification effect of the model through the best combination of features. Finally, methods such as the Silhouette Coefficient or Elbow Method are used to determine the optimal number of clusters (*k*) [47, 48].

**Fig 3:**
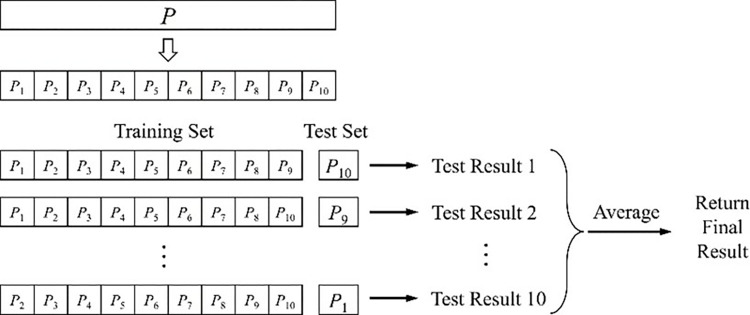
Schematic diagram of 10-fold cross-validation.

In addition, k-fold cross-validation is used to assess the robustness and generalization ability of the model [49]. The dataset is divided into *k* subsets, and one of the subsets is used as the validation set each time, and the remaining *k*-1 subsets are used as the training set, and repeated k times. Calculate the evaluation metrics for each validation and take the average as the final performance of the model (Fig 3).

### 2.5 Evaluation index

After performing land cover classification of high-resolution remote sensing images using the spectral clustering algorithm, it is crucial to evaluate the quality of the classification results. The following are two commonly used evaluation metrics:

Overall accuracy measures the overall correctness of the classification model and is calculated as follows [50]:

$$OA=\frac{\sum_{i=1}^{k}n_{ii}}{N}$$ (11)

Where, *n*_*ii*_ represents the diagonal elements of the confusion matrix, indicating the number of pixels of the *i*-th class that are correctly classified. *N* is the total number of pixels. Overall accuracy reflects the classification accuracy of all categories, but it does not reflect the specific performance of each category.

The Kappa coefficient is used to measure the consistency of the classification results relative to the results of random assignment [51], taking into account the correct classifications that occur by chance. The formula for calculation is:

$$Kappa=\frac{\begin{array}{l} p_{o}-p_{e} \end{array}}{1-p_{e}}$$ (12)

Where, *p*_*o*_​ represents the actual agreement, which is the proportion of correct classifications; *p*_*e*_ represents the expected agreement, which is the expected proportion of random classifications. The value of the Kappa coefficient ranges from -1 to 1, where 1 indicates perfect agreement, 0 indicates random agreement, and negative values indicate that the classification results are worse.


$$SE(Kappa)=\sqrt{\frac{\begin{array}{l} p_{o}\times (p_{o}-p_{e}) \end{array}}{N\times {\left(1-p_{e}\right)}^{2}}}$$ (13)

$$Z=\frac{\begin{array}{l} Kappa \end{array}}{SE(Kappa)}$$ (14)

P−Value=2•(1−Φ(|Z|)) (15)

Where, *SE*(*Kappa*) represents the standard error, Φ(|*Z*|) denotes the cumulative distribution function (CDF) of the standard normal distribution for a two-tailed test, and *Z* refers to the test statistic.

## 3. Results and discussion

### 3.1 Feature extraction

**Fig 4:**
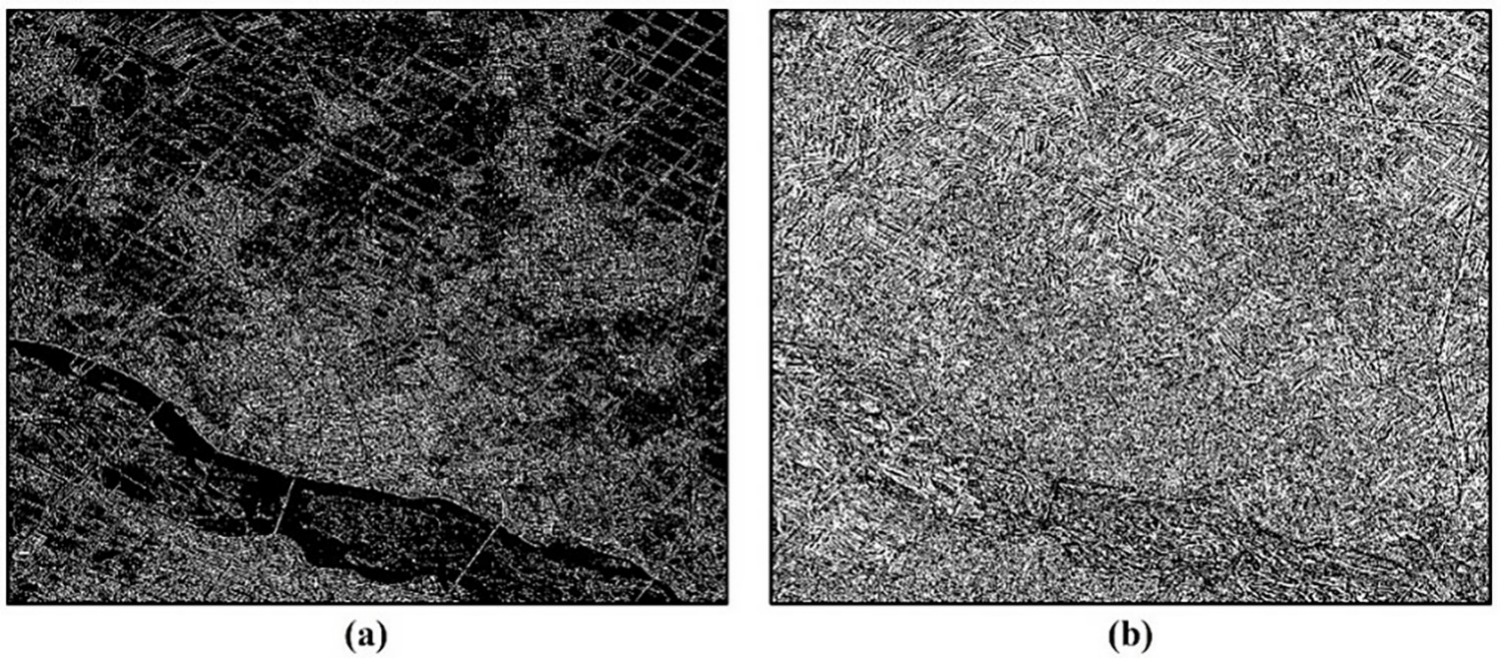
Laplacian filtered images: (a) the size of filter is 3*3 (b) the size of filter is 7*7.

Due to the complexity of land cover types in the area, in addition to extracting the spectral information from high-resolution remote sensing imagery, classification is also performed using its shape and texture information, which can address the phenomenon of "same object, different spectra". In remote sensing imagery, there are clear boundaries between different types of land features. Utilizing edge shape information can enhance the classification accuracy of remote sensing images. In this study, Laplacian high-pass filtering is used for filtering treatment, generating images with grayscale ranging from 0 to 255, with filter sizes of 3x3 and 7x7, respectively. The images processed by the Laplacian algorithm are shown in Fig 4. Edge information is extracted by setting thresholds through histogram analysis, generating binary images of edge density.

**Fig 5:**
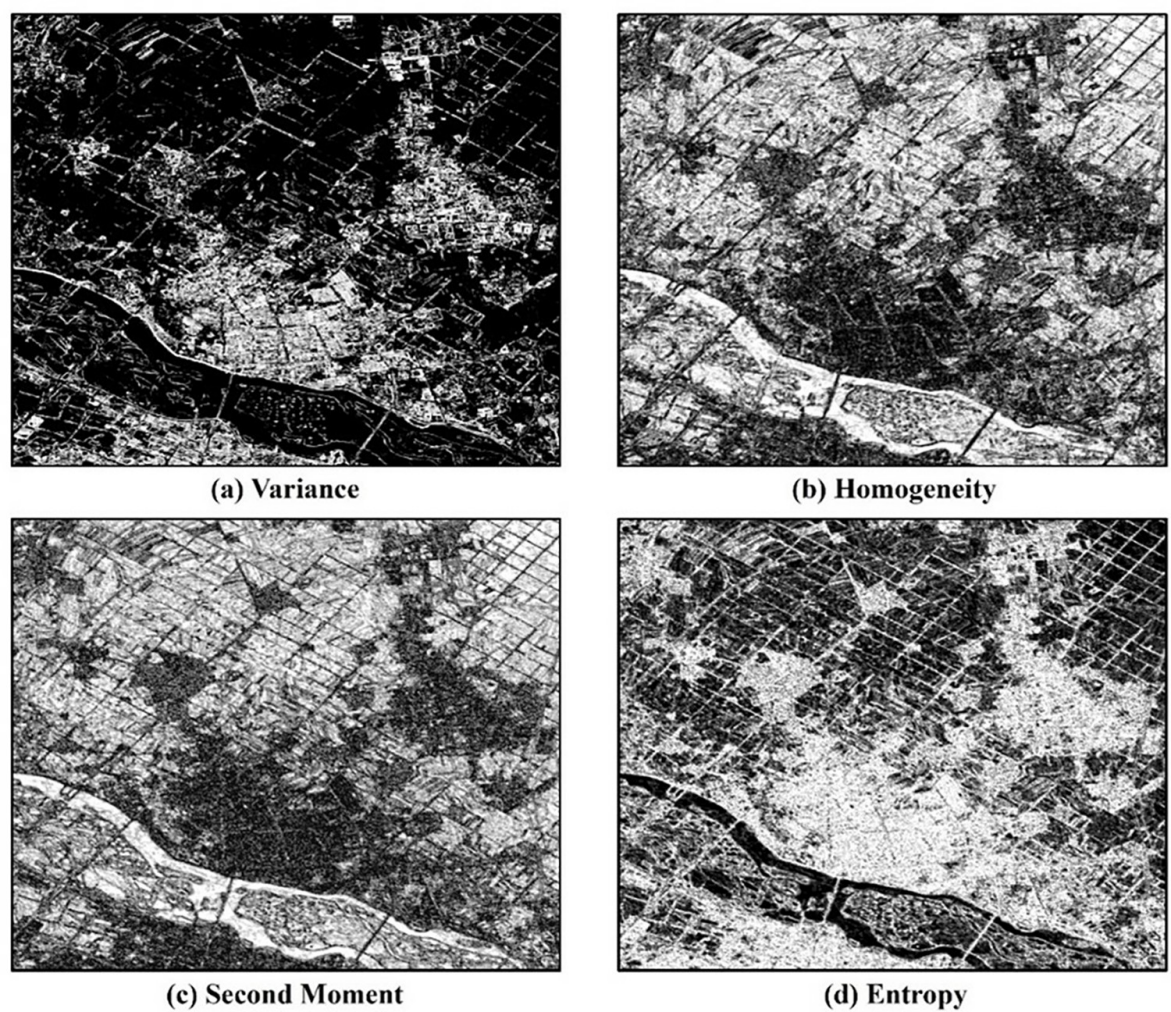
Image texture features: (a) variance (b) homogeneity (c) second moment (d) entropy.

The Gray-Level Co-occurrence Matrix (GLCM) proposed by Haralick is a classic in statistical methods (Haralick et al., 1973). The GLCM method calculates the occurrence frequency of each gray level at a specified direction and distance, generating the corresponding GLCM. Then, second-order statistical feature values are computed as texture measures to describe the image. The GLCM defines the calculation methods for different texture features. By combining spectral features, texture features are extracted using variance, uniformity, entropy, and second-order moments (Fig 5).

### 3.2 Classification and accuracy verification

To validate the applicability of the spectral clustering optimized by the Lanczos algorithm in the classification of high-resolution remote sensing imagery, the images of the study area were classified using KNN, SC, and SNN-MSC algorithms, and a land cover map of the study area was produced. In this study, the parameter *c* in the K-means algorithm represents the actual number of clusters, while the parameter *c* in the SC algorithm denotes the number of clusters. Extensive experiments have demonstrated that the SNN-MSC algorithm performs optimally when the parameter *k* is set between 3 and 20, and the parameter *θ* is set between 1 and 10.

**Fig 6:**
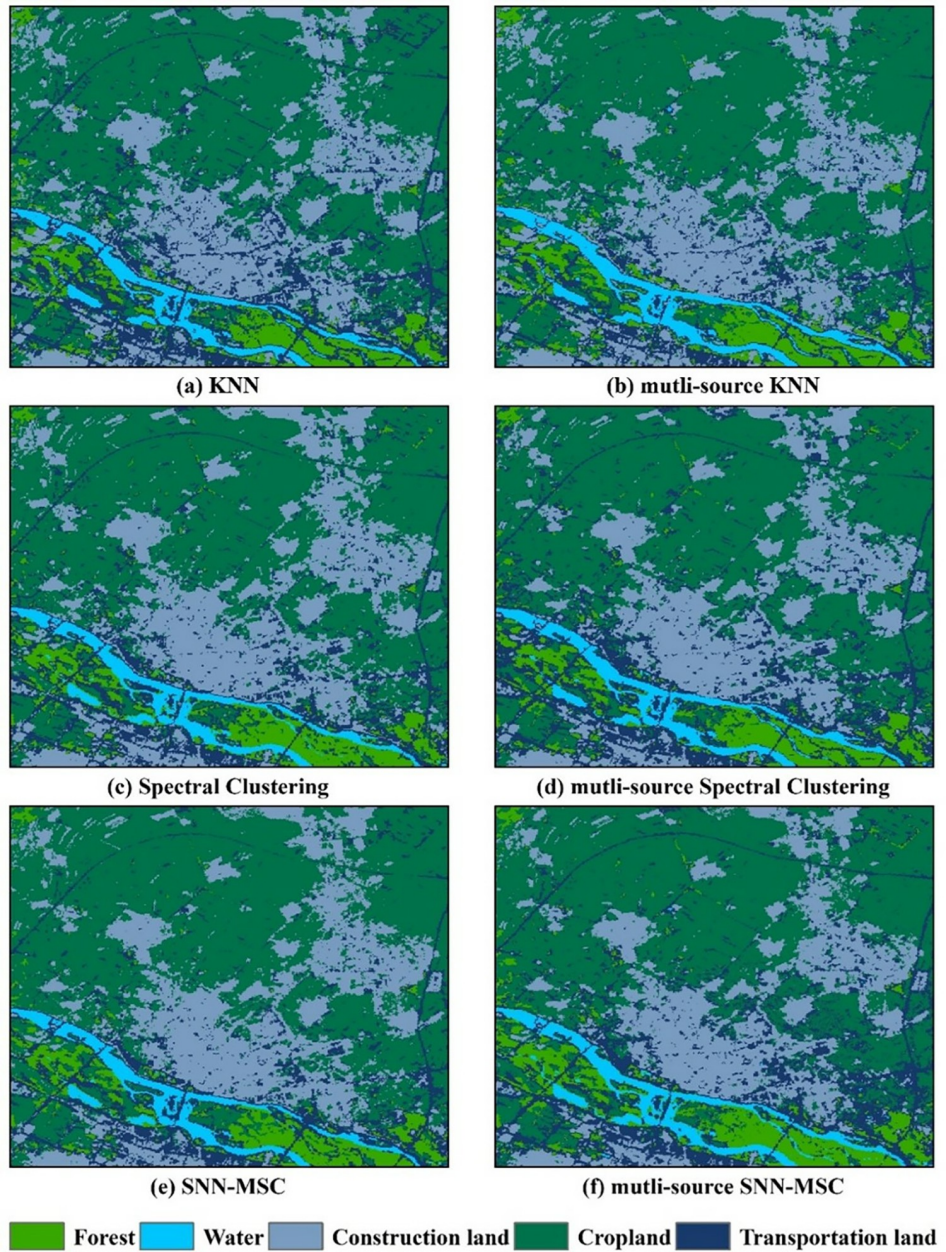
Land cover classification results: (a) ISODATA, (b) mutli-source ISODATA, (c) k-means, (d) multi-source k-means, (e) spectral clustering, and (f) multi-source spectral clustering.

Additionally, classification experiments were conducted considering only spectral information, edge shape, and texture information, which were randomly divided into training groups (model establishment and parameter optimization) and validation groups (model accuracy and generalization ability) in a 3:1 ratio, testing the effect of multi-source information on high-resolution image classification. The results are shown in Fig 6. As shown in Fig 6, both the KNN and SC algorithms, when considering only spectral information, exhibit some missing and incorrect classifications, particularly in areas with sharp grayscale changes (such as the boundary between vegetation and water bodies). However, when edge shape and texture information are incorporated, the model’s classification performance shows a significant improvement. According to the recognition results in Fig 6(E) and 6(F), SNN-MSC outperforms the other two algorithms when considering only spectral information and when using multi-source information. The performance improvement is especially noticeable in the case of multi-source information-based quantitative classification. This indicates that the SNN-MSC algorithm, when considering multi-source information, can effectively identify boundaries between linear objects and different textures, providing a more precise delineation of the boundary between vegetation and water bodies compared to the other two methods. Additionally, for land cover types with similar spectral and texture characteristics, such as construction land and transportation land, SNN-MSC achieves higher accuracy, significantly reducing classification errors and omissions. Therefore, SNN-MSC exhibits more robust adaptability to data distribution and better clustering performance.

### 3.3 Performance evaluation

This study utilizes a confusion matrix to analyze the accuracy of classification results. Using ZY1-02D imagery as validation data, a spatially balanced sampling method was applied to randomly select 300–500 sample points for each land cover type. The accuracy of the aforementioned methods is assessed by calculating the classification confusion matrix to obtain overall accuracy, user’s accuracy, producer’s accuracy (PA), and the Kappa coefficient. The classification results are statistically presented in Table 1, and a comparison of classification accuracy indicators is shown in Table 2. As illustrated in Table 1, the overall classification accuracy for water bodies and residential areas is relatively high, while the classification accuracy for forests is generally not high. When classifying using pure spectral information, shape information is utilized to delineate the boundaries of land features. After incorporating shape information, the classification accuracy of the three algorithms has significantly improved, with an average increase of 13.89%. As shown in Table 2, the SNN-MSC algorithm exhibits higher classification accuracy for different land cover types compared to the previous KNN and SC algorithms. The overall classification accuracy is 84.63%, and the Kappa coefficient is 0.846, validating the efficiency and superiority of spectral clustering optimized by the Lanczos algorithm in the classification of high-resolution remote sensing imagery. The classification accuracy of multi-source information combined with shape and texture is significantly higher than that of spectral information alone. The Kappa coefficient has improved by 7.7%, 19.11%, and 14.93%, respectively.

**Table 1:** Classification accuracy of the spectral clustering (in %).

Classified data	Reference Data				
	Water	Transportation land	Construction land	Forest	Cropland
**Water**	88.03	1.52	2.03	5.93	1.27
**Transportation land**	0	85.29	0.57	3.96	2.31
**Construction land**	4.54	2.81	86.44	5.39	8.75
**Forest**	1.22	0	6.91	80.96	5.24
**Cropland**	6.21	10.38	4.05	3.76	82.43

**Table 2:** Comparisons of classification accuracy indicators.

Classified method	User accuracy (%)					Overall accuracy (%)	Kappa coefficient(%)	P-Value
	Water	Transportation land	Construction land	Forest	Cropland			
**KNN**	53.59	48.38	55.62	45.52	51.76	51.77	51.68	0.0954
**SC**	58.46	51.68	63.03	50.77	54.32	55.65	55.56	0.0856
**SNN-MSC**	75.82	70.87	74.31	61.99	65.54	69.71	69.66	0.0159
**Multi-source KNN**	62.42	60.35	63.73	51.01	59.83	59.46	59.38	0.0799
**Multi-source SC**	76.43	73.87	78.44	72.31	72.56	74.72	74.67	0.0081
**Multi-source SNN-MSC**	88.03	85.29	86.44	80.96	82.43	84.63	84.59	0.0089

**Fig 7:**
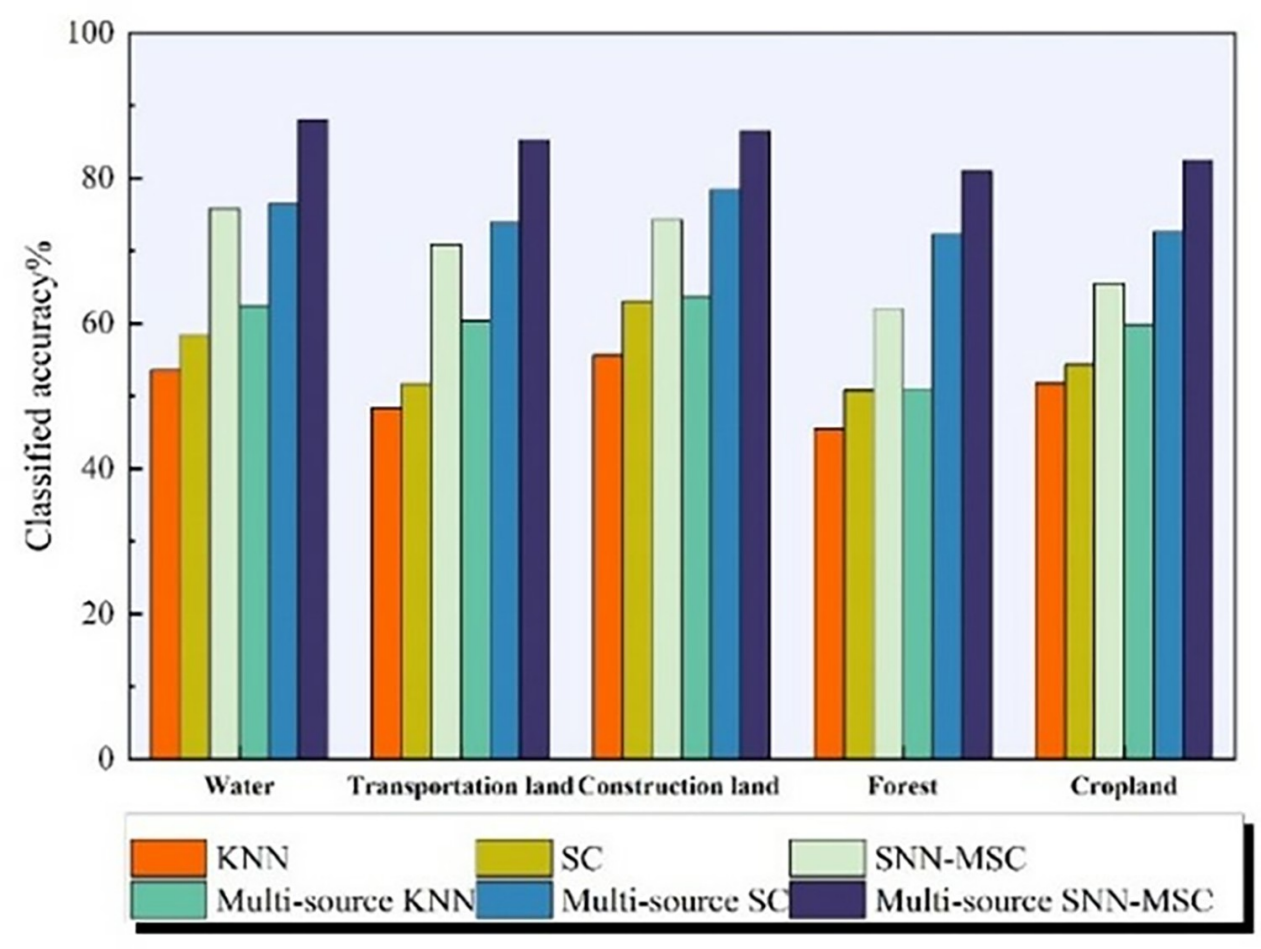
Comparison of PAs.

The comparison of Producer’s Accuracy (PA) among several experiments is depicted in Fig 7. The figure indicates that the SNN-MSC with multi-source information achieved a higher Kappa coefficient than other methods. Notably, when using pure spectral classification, the classification accuracy for transportation land is relatively low. However, after incorporating shape and texture features, the accuracy for transportation land has significantly improved compared to other land use types, reaching an increase of 14.42%. The significance test results indicate that the p-values of SNN-MSC under both single data source and multi-source information conditions are less than 0.05, demonstrating that it has passed the significance test. This finding suggests that the Kappa coefficient is statistically significant, allowing for rejecting the null hypothesis and indicating that SNN-MSC exhibits a high level of consistency under both conditions.

**Fig 8:**
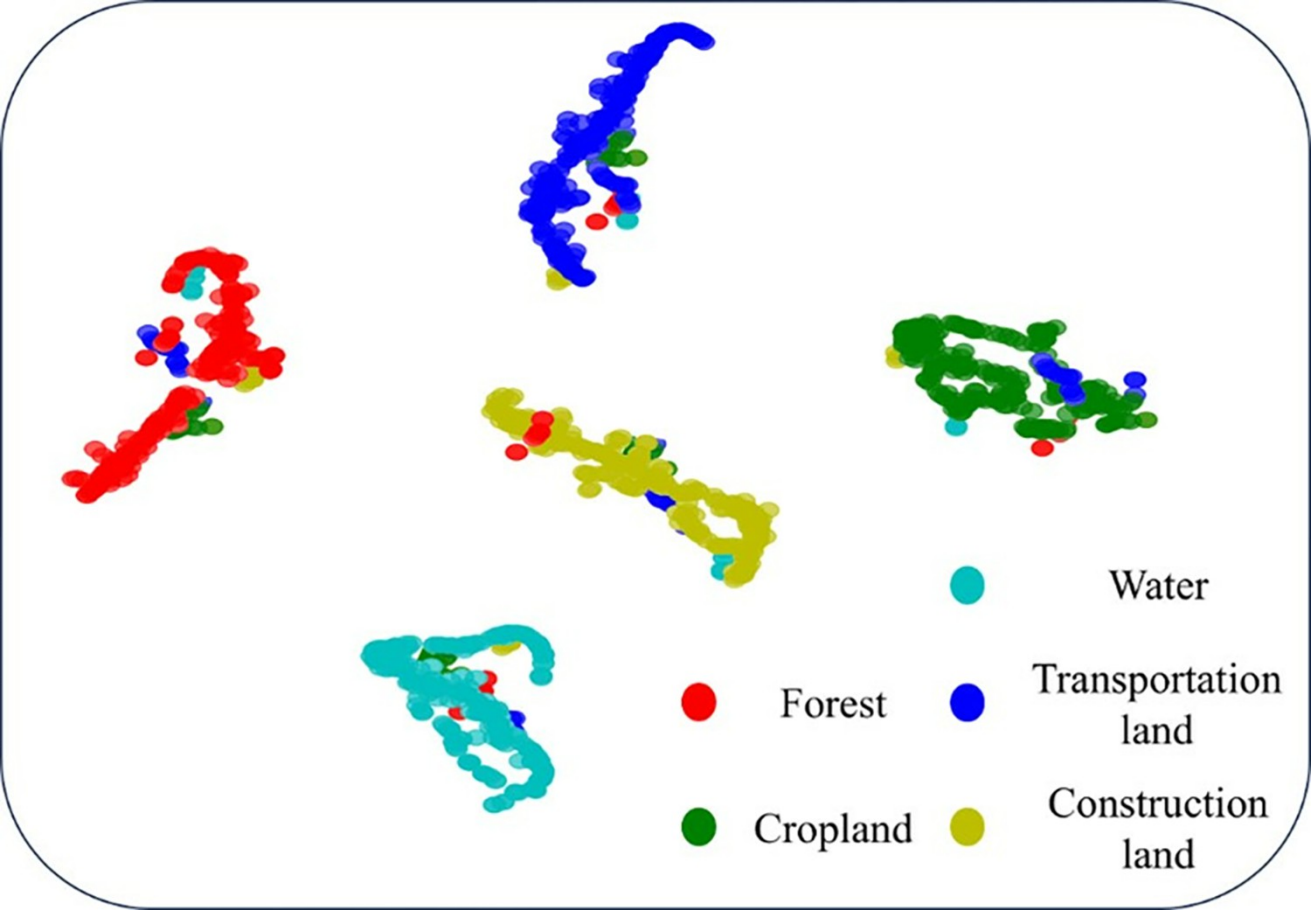
Visualization of learned features via t-SNE.

To visually demonstrate the superiority of SNN-MSC in clustering tasks, the t-SNE technique was employed for feature visualization. Fig 8 presents the visualization results of the features learned in the last layer mapped to a two-dimensional space. From Fig 8, it can be observed that there are clear boundaries in the feature space of the five land classes, with a small number of feature points being confused between different classes, and a phenomenon of feature homology is exhibited in the forest and transportation land. This is consistent with the analysis results in Table 1 and Fig 6, providing corroboration for the high performance of SNN-MSC.

### 3.4 Complexity analysis of the SNN-MSC algorithm

This section provides a complexity analysis of the SNN-MSC algorithm, where *n* denotes the number of samples, *d* represents the sample dimensionality, and *k* is the number of nearest neighbors. The detailed analysis process is as follows:

1. The time complexity for calculating Euclidean distance in distance measurement is *O*(*dn*^2^), the time complexity for computing the exponential term is *O*(*n*), and the time complexity for the density factor is *O*(*kn*).
2. In this study, the shortest path distance calculation is implemented using Dijkstra’s algorithm, with a time complexity of *O*(*n*^2^) [52].
3. The time complexity for calculating similarity between samples is *O*(*n*^2^).
4. The time complexity for computing the normalized similarity matrix is *O*(*n*^2^).
5. The time complexity for calculating *r*_1_,*r*_2_,⋯*r*_*n*_​ and *r* is *O*(*kn*).
6. The eigen decomposition of the Laplacian matrix is performed using Singular Value Decomposition (SVD), with a time complexity of *O*(*n*^3^).
7. The time complexity for updating the similarity matrix *S* is *O*(*n*^2^).

In summary, the overall time complexity of the SNN-MSC algorithm is *O*(*n*^3^), which is of the same order of magnitude as that of the SC algorithm.

## 4. Conclusions

This study applies the SNN-MSC algorithm to remote sensing image processing, providing a novel approach for unsupervised classification of remote sensing images. Compared to previous spectral clustering optimization methods, this approach integrates spectral clustering with a density factor and replaces traditional distance metrics, such as Euclidean distance, with manifold distance. Adjusting the exponential term and scaling factor simultaneously satisfies both global and local consistency, better uncovering the intrinsic structure of the data. Furthermore, by utilizing shared neighbor information between samples and redefining the similarity measure through an exponential kernel, the method, combined with a rank constraint applied to the Laplacian matrix, enables adaptive graph learning, leading to a more accurate capture of the true data structure.

Based on ZY1-02D remote sensing imagery, the classification performance of the SNN-MSC algorithm was validated for land cover classification. The results indicate that, compared to using only spectral information, the SNN-MSC algorithm combined with multi-source information achieves an overall classification accuracy of 84.63%, with a 14.93% increase in the Kappa coefficient. Additionally, the SNN-MSC algorithm with multi-source information achieves producer accuracies (PA) for water bodies, transportation land, construction land, forest, and farmland of 88.03%, 85.29%, 86.44%, 80.96%, and 82.43%, respectively, significantly outperforming other comparative algorithms. The feature visualization results also provide favorable evidence supporting these findings. This demonstrates that the SNN-MSC algorithm, when combined with high-resolution remote sensing data, can quickly perform land cover classification, offering a feasible solution that enhances the accuracy and efficiency of land cover type identification. Furthermore, it shows a notable improvement in accuracy, particularly when handling complex-shaped data, such as transportation land, compared to other widely used methods.
